# Dynamics of the Fermentation Products, Residual Non-structural Carbohydrates, and Bacterial Communities of Wilted and Non-wilted Alfalfa Silage With and Without *Lactobacillus plantarum* Inoculation

**DOI:** 10.3389/fmicb.2021.824229

**Published:** 2022-01-11

**Authors:** Fengyuan Yang, Yanping Wang, Shanshan Zhao, Changsong Feng, Xiaomiao Fan

**Affiliations:** ^1^Henan Provincial Key Laboratory of Ion Beam Bio-Engineering, School of Physics, Zhengzhou University, Zhengzhou, China; ^2^Henan Provincial Key Laboratory of Ion Beam Bio-Engineering, School of Agricultural Science, Zhengzhou University, Zhengzhou, China; ^3^Institute of Animal Husbandry and Veterinary Science, Henan Academy of Agricultural Sciences, Zhengzhou, China

**Keywords:** absolute quantification 16S-seq, alfalfa silage, bacterial community, moisture, non-structural carbohydrates dynamics

## Abstract

The aim of this study was to investigate effects of wilting and *Lactobacillus plantarum* inoculation on the dynamics of the fermentation products, residual non-structural carbohydrates, and bacterial communities in alfalfa silage. Fresh and wilted alfalfa were ensiled with and without *L. plantarum* for 10, 30, 60, and 90 days. A high-throughput sequencing method for absolute quantification of 16S rRNA was adopted to determine the bacterial community composition at different ensiling periods. For the wilted silage, the bacterial community, pH value, and ammonia nitrogen concentration remained stable in the silage at 30 days. *L. plantarum* inoculation accelerated lactic acid fermentation and altered the predominant genus in the wilted silage as compared with the non-inoculated group. For the non-wilted group, fast consumption of water-soluble carbohydrates (WSCs) was observed at 10 days in the non-inoculated silage along with rapid growth of undesirable *Hafnia*. *L. plantarum* inoculation inhibited growth of *Hafnia* at 10 days in the non-wilted silage. Clostridia fermentation occurred in the non-wilted silage at 90 days, as indicated by an increased pH, formation of butyric acid (BA), and apparent abundance of genera belonging to Clostridia. *L. plantarum* inoculation inhibited BA accumulation and growth of *Garciella* in the non-wilted silage at 90 days as compared with the non-wilted silage without inoculation, but had little effect on the growth of *Clostridium sensu stricto*. Overall, the high moisture content of the non-wilted alfalfa silage led to rapid consumption of WSCs and growth of harmful microorganisms at the early stage of ensiling, resulting in poor fermentation quality. Wilting and *L. plantarum* inoculation both improved fermentation quality and inhibited the growth of spoilage microorganisms in alfalfa silage, while *L. plantarum* inoculation alone failed to achieve optimum fermentation quality of non-wilted alfalfa silage.

## Introduction

Year-round access to good quality silage is essential for ruminants. As forage production is seasonal in many areas, ensiling is used globally for forage preservation, especially during rainy seasons (Eikmeyer et al., 2013). In an optimum ensiling process, lactic acid (LA) bacteria (LAB) rapidly convert water-soluble carbohydrates (WSCs) into organic acids, mainly LA, and outcompete other microorganisms to dominate the bacterial community. However, the competition of LAB against other microorganisms is affected by multiple factors, including moisture content, aerobic conditions, and composition of epiphytic microorganisms in fresh forage (Eikmeyer et al., 2013; Kasmaei et al., 2017; Wang et al., 2019).

Alfalfa is a perennial legume with a high nutritional value that is commonly applied for ensiling worldwide. *Lactobacillus plantarum* is the most commonly used bacterial inoculant in forage ensiling studies (Oliveira et al., 2017) and is also widely applied in alfalfa silage production. The moisture content of alfalfa plays an important role in ensiling fermentation. For wet grasses and legumes like alfalfa, wilting to an appropriate dry matter (DM, 300–400 g/kg fresh weight (FW)] is recommended to prevent effluent production (Duniere et al., 2013). Previous studies have reported an increased risk of Clostridia fermentation when the moisture content of the alfalfa silage exceeds 70% (Coblentz and Muck, 2012; Yang et al., 2020). Previous studies reported that wilting of wet alfalfa material before ensiling could improve the fermentation quality of the silage (Tao et al., 2017; Agarussi et al., 2019). However, the effects of wilting on bacterial community dynamics of alfalfa silage remain unclear. Besides, alfalfa is generally considered difficult to ensile due to a high buffering capacity and low concentration of WSCs. As reported by Tao et al. (2017) and Agarussi et al. (2019), although wilting reduces buffering capacity, the WSC concentration in wilted alfalfa material is also reduced, which could affect the ensiling process. The abundance of epiphytic microbes might also be altered along with the consumption of WSCs during wilting.

Application of next-generation sequencing has been key to elucidate the roles of microbes in the alfalfa ensiling process (Guo et al., 2018; Ogunade et al., 2018; Yang et al., 2019, 2020). While conventional next-generation sequencing can clearly identify microbes in a single sample, it does not reflect differences in the abundance of microbes across samples (Smets et al., 2016). Previous studies have indicated that wilting may affect the abundance of epiphytic microbes and *L. plantarum* as inoculants in alfalfa silage (Tao et al., 2017; Agarussi et al., 2019). Maintaining an absolute abundance is, thus, necessary to explore effects of wilting and *L. plantarum* inoculation on the dynamics of bacterial communities. An absolute quantitation method was recently developed using synthetic chimeric DNA spikes (Tkacz et al., 2018) and was successfully implemented to compare the abundances of different microbial taxa across soil, water, and silage samples (Jiang et al., 2019; Tan et al., 2020; Yang et al., 2021).

The objective of this study is to investigate effects of wilting on the dynamics of fermentation products, residuals of non-structural carbohydrates, and bacterial communities in alfalfa silage with and without *L. plantarum* inoculation. The proposed absolute quantification method was applied to clarify the comprehensive dynamics of bacterial communities during the ensiling process.

## Materials and Methods

### Silage Preparation

Alfalfa was grown on a research farm operated by the Henan Academy of Agricultural Sciences (Zhengzhou, China). Fresh alfalfa in the early bloom stage was harvested at 5 cm above ground level. For the non-wilted group, fresh alfalfa was chopped into pieces at 1–2 cm in length, while for the wilted group, alfalfa was wilted in the field to a DM of 371.57 g/kg FW and then chopped. The following treatments were formed for analysis: HM, not wilted before ensiling; MM, wilted before ensiling; CK, silage without inoculation; and LP, silage inoculated with *L. plantarum* A345. *L. plantarum* A345 is an alfalfa epiphytic strain isolated from Shanxi, China (Yang et al., 2020). Both wilted and non-wilted alfalfa were treated with (1) distilled water as a control or (2) 1 × 10^6^ colony-forming units/g of *L. plantarum*. Three replications were performed for each treatment at each of the four ensiling periods. Thus, for each treatment, 12 kg of alfalfa was randomly divided into 24 equal parts (500 g each) and packed into polyethylene plastic bags, which were vacuum sealed with a Shineye P-290 Vacuum Packaging Sealer (Dongguan Yijian Packaging Machinery Co., Ltd., Dongguan, China). The silage samples were stored at room temperature of 24–28°C for 10, 30, 60, and 90 days.

### Analysis of Fermentation Products

A 10-g sample from each bag was mixed with 90 ml of sterilized water by shaking at 160 rpm for 1 h at 4°C using a shaker (JBXL-70, Putian Co., Ltd., Changzhou, China) and then filtered through a 0.45-μm membrane. The pH value was determined using a glass electrode pH meter (Mettler-Toledo, GmbH, Greifensee, Switzerland). The organic acid content [i.e., LA, acetic acid (AA), propionic acid (PA), and butyric acid (BA)] was determined by high-performance liquid chromatography (Waters Corporation, Milford, MA, United States) in accordance with the procedure described by Zhao et al. (2020). The concentration of ammoniacal nitrogen (NH_3_-N) was determined using the Berthelot colorimetric test (Broderick and Kang, 1980).

A sample of approximately 150 g from each bag was dried in an oven for 48 h at 65°C (AOAC, 1990), then pulverized using a pulverizer (COSUAI CS-2500, Wuyi Haina Electric Appliance Co., Ltd., Jinhua, China) and passed through a 1-mm screen to determine the WSC and monosaccharide compositions. The WSC concentration was determined using the anthrone-sulfuric acid colorimetric assay (Murphy, 1958), while the monosaccharide composition (i.e., glucose, fructose, and galactose) was determined using a ICS-3000 Ion Chromatography System with an Analytical CarboPac PA10 pellicular anion-exchange resin column (Dionex, Sunnyvale, CA, United States) and an amperometric detector in accordance with the procedure described by Guo et al. (2019). The monosaccharide was eluted with 25 mM NaOH with the flow rate of 1.0 mL/min.

### Bacterial Community Analysis

A 10-g sample from each bag was mixed with 100 ml of sterile phosphate-buffered saline (pH 7.2) by shaking at 160 rpm for 2 h at 4°C using a shaker (JBXL-70, Putian Co., Ltd., Changzhou, China), and then filtered through four layers of cheesecloth. The liquor was then centrifuged at 8000 rpm for 15 min at 4°C. The precipitate was resuspended in 1 ml of sterile phosphate-buffered saline. The microbe pellet was collected by centrifugation at 12000 rpm for 2 min at 4°C. Total DNA was extracted with an E.Z.N.A.^®^ Bacterial DNA Kit (D3350-02; Omega Bio-Tek, Inc., Norcross, GA, United States) in accordance with the manufacturer’s instructions. The V3–V4 region of 16S rRNA was amplified (approximately 460 bp) using the forward Primer F (Illumina adapter sequence 1 + CCTACGGGNGGCWGCAG) and the reverse Primer R (Illumina adapter sequence 2 + GACTACHVGGGTATCTAATCC) (Illumina, Inc., San Diego, CA, United States). *Escherichia coli* CMCC (B) 44102 (NRRL accession No. B-1109) was used as a positive control to confirm that the procedure correctly assessed this identification and that the primers used worked normally. Absolute quantification 16S-seq was conducted using an Illumina MiSeq PE250 sequencer (Illumina, Inc.) by Genesky Biotechnologies Inc. (Shanghai, China) following the procedure described by Smets et al. (2016) and Tkacz et al. (2018). Briefly, the variable regions of synthetic chimeric DNA spikes were designed to be lacking identity to nucleotide sequences deposited in public databases. These spikes can be used as internal standards for absolute quantification with known amounts of spikes added to the samples. In the current study, nine synthetic chimeric DNA spikes were used as internal standards. Spikes were added to the sample DNA pools at four concentrations (10^3^, 10^4^, 10^5^, and 10^6^ copies of internal standards). The PCR products were purified with Agencourt AMPure XP nucleic acid purification magnetic beads (Beckman Coulter, Inc., Brea, CA, United States). The synthetic chimeric DNA spikes were filtered out and raw reads were checked using FLASH2 (Fast Length Adjustment of SHort reads; version 2.2.00).^1^ High-quality sequences were clustered into operational taxonomic units (OTUs) using Uparse (version 7.0.1001)^2^ at 97% similarity. Taxonomy assignment of representative sequences was performed using the Ribosome Database Project (Cole et al., 2009). The alpha diversity indices of bacterial communities were calculated using mothur (version 1.9.0).^3^ Linear discriminant analysis effect size (LEfSe) analysis was performed using python (version 2.7.14).^4^

### Data Accessibility

The sequences were archived in the Sequence Read Archive with the accession number PRJNA773516.

### Statistical Analyses

Experimental data of the fermentation products, residual non-structural carbohydrates, and alpha diversity indices of bacterial communities were analyzed with a general liner model using IBM SPSS Statistics for Windows, version 21.0. (IBM Corporation, Armonk, NY, United States). The effects of different treatments were evaluated by one-way analysis of variance followed by Duncan’s multiple range test. Spearman’s correlation coefficients were generated using the R software (version 2.15.3).^5^ A probability (*P*) value of <0.05 was considered statistically significant.

## Results

### Characteristics of Wilted and Non-wilted Alfalfa

The fresh alfalfa had a pH of 6.35, DM of 237.15 g/kg FW, and WSC, glucose, fructose, and galactose concentrations of 82.06, 8.81, 14.02, and 1.15 g/kg DM, respectively. The wilted alfalfa had a pH of 6.57, DM of 371.51 g/kg FW, and WSC, glucose, fructose, and galactose concentrations of 76.11, 9.19, 11.19, and 0.93 g/kg DM, respectively. No formation of organic acids or NH_3_-N was detected in the wilted and non-wilted alfalfa.

### Effects of *Lactobacillus plantarum* on the Fermentation Properties of Wilted and Non-wilted Alfalfa Silage

As shown in Table 1, wilting, inoculation, days of ensiling, and the interactions of these factors had significant effects on the pH, NH_3_-N content, and LA and AA concentrations of alfalfa silage (*P* < 0.05), with only slight effects of inoculation on the AA concentration (*P* = 0.09), while the interactions of wilting, inoculation, and days of ensiling had no effect on the NH_3_-N content (*P* = 0.26).

**TABLE 1:** Effects of wilting (W), inoculation (I), ensiling days (D), and the interactions of these factors on the fermentation properties and non-structural carbohydrates of alfalfa silage.

	W	I	D	W × I	W × D	I × D	W × I × D
***Fermentation properties* ^1^**							
pH	***	***	***	**	***	***	***
NH_3_-N	***	***	***	***	***	*	NS
LA	***	***	***	***	***	***	***
AA	***	NS	***	**	**	**	***
***Non-structural carbohydrates* ^2^**							
WSC	***	*	***	NS	***	***	**
Glucose	***	*	***	NS	***	***	***
Fructose	***	***	***	***	***	***	***
Galactose	***	**	***	***	**	*	NS

*P < 0.05; **P < 0.01; ***P < 0.001. NS, not significant.

*^1^NH_3_-N, ammoniacal nitrogen; LA, lactic acid; AA, acetic acid.*

*^2^WSC, water soluble carbohydrate.*

**FIGURE 1:**
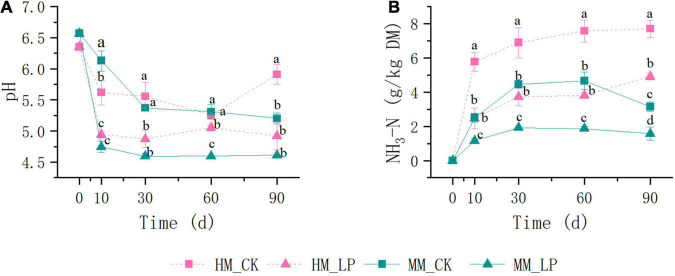
Dynamics of pH **(A)** and NH_3_-N **(B)** concentration during ensiling. HM, not wilted before ensiling; MM, wilted before ensiling; CK, silage without inoculation; LP, silage inoculated with *L. plantarum* A345. Means with different small letters represents significant difference among treatments at the same ensiling period at *P* < 0.05 (*n* = 3, bars indicate standard error of means).

**FIGURE 2:**
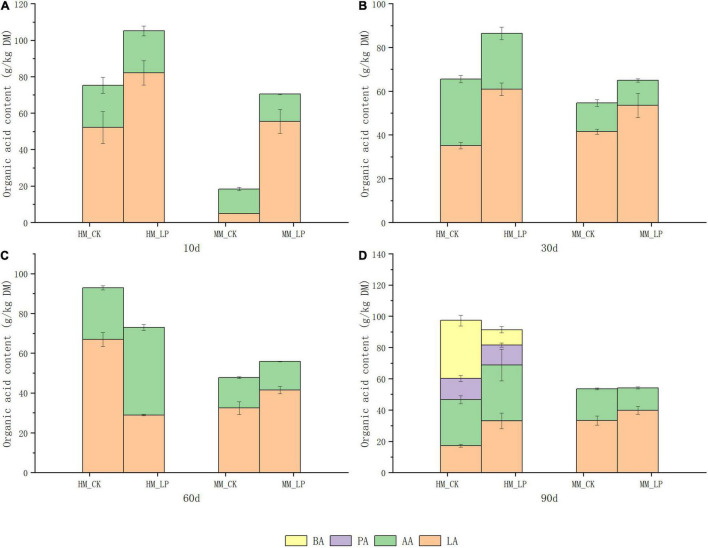
Accumulation of organic acids at 10 **(A)**, 30 **(B)**, 60 **(C)**, and 90 **(D)** days. HM, not wilted before ensiling; MM, wilted before ensiling; CK, silage without inoculation; LP, silage inoculated with *L. plantarum* A345.

Wilting prior to ensiling promoted decreases in pH and NH_3_-N accumulation in alfalfa silage (*P* < 0.05, Figure 1), and prevented the apparent formation of PA and BA at 90 days (*P* < 0.05, Figure 2). Inoculation of *L. plantarum* promoted decreases in pH and NH_3_-N accumulation as compared with the CK wilted and non-wilted groups (*P* < 0.05). Inoculation also accelerated LA accumulation in both wilted and non-wilted silages at 10 and 30 days (*P* < 0.05, Figure 2), but had little effect on the AA concentration throughout the entire ensiling process. In the non-wilted silage, inoculation of *L. plantarum* inhibited BA formation at 90 days as compared with the CK group (*P* < 0.05), but had little effect on PA accumulation at 90 days in the non-wilted silage.

### Effects of *Lactobacillus plantarum* on Residual Non-structural Carbohydrates in the Wilted and Non-wilted Alfalfa Silages

Effects of wilting, inoculation, days of ensiling, and the interactions of these factors on residual non-structural carbohydrates are shown in Table 1. These three factors as well as the interactions had significant effects on WSC, glucose, fructose, and galactose concentrations in alfalfa silage (*P* < 0.05), except that the interaction of wilting and inoculation had only a slight effect on the WSC (*P* = 0.11) and glucose (*P* = 0.13) concentrations, while the interaction of wilting, inoculation, and days of ensiling had no effect on the galactose concentration (*P* = 0.65).

**FIGURE 3:**
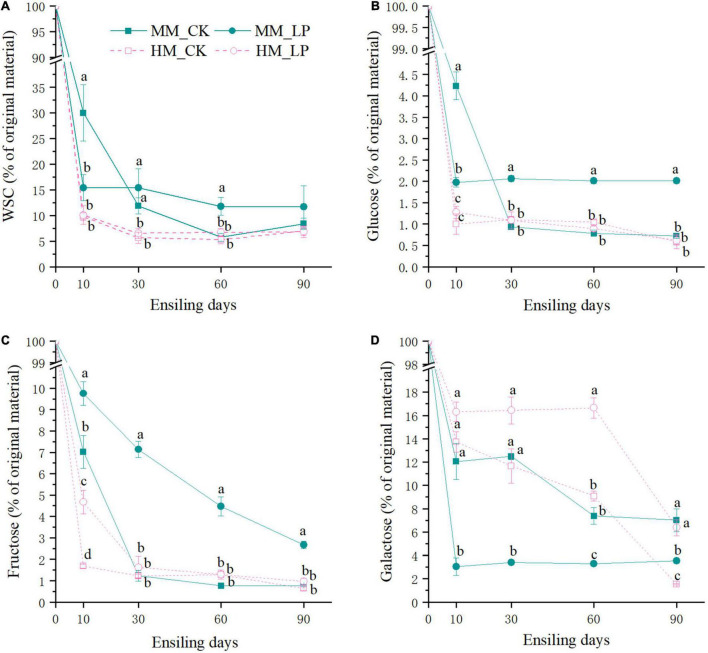
Dynamics of residual WSC **(A)**, glucose **(B)**, fructose **(C)**, and galactose **(D)** during ensiling and expressed as percentage in the parent material. Compositions of WSC and monosaccharide in the non-wilted and wilted alfalfa prior to ensiling served as 100%. HM, not wilted before ensiling; MM, wilted before ensiling; CK, silage without inoculation; LP, silage inoculated with *L. plantarum* A345. Means with different small letters represents significant difference among treatments at the same ensiling period at *P* < 0.05 (*n* = 3, bars indicate standard error of means).

The wilted group had higher residual contents of WSC, glucose, and fructose at 10 days in the non-inoculated silage as compared with the non-wilted group (*P* < 0.05, Figure 3). The inoculated silage had a higher residual content of fructose at 10 days and maintained higher residual contents of galactose at 60 and 90 days as compared with the non-inoculated silage in the non-wilted group (*P* < 0.05). At 90 days, the residual content of galactose was significantly reduced in the non-wilted silage as compared with the 60-day silage (*P* < 0.05). In the wilted silage, inoculation of *L. plantarum* enhanced the reduction in WSC and glucose concentrations at 10 days and promoted glucose maintenance afterward as compared with the CK group (*P* < 0.05). Inoculation of *L. plantarum* increased consumption of galactose and decreased consumption of fructose as compared with the non-inoculated silage in the wilted group (*P* < 0.05).

### Effects of *Lactobacillus plantarum* on Bacterial Communities of Wilted and Non-wilted Alfalfa Silages

In total, 13923372 reads were acquired for bacterial community analyses of 54 samples (4 treatments × 4 ensiling periods × 3 replications + 3 non-wilted alfalfa samples + 3 wilted alfalfa samples), with spike-in reads accounting for 28.77% ± 6.73%. These valid sequences were clustered into 1029 OTUs based on a 97% sequence identity. The number of gene copies per ng of DNA was calculated from standard curves with fitting coefficients (*R*^2^) > 0.99.

**FIGURE 4:**
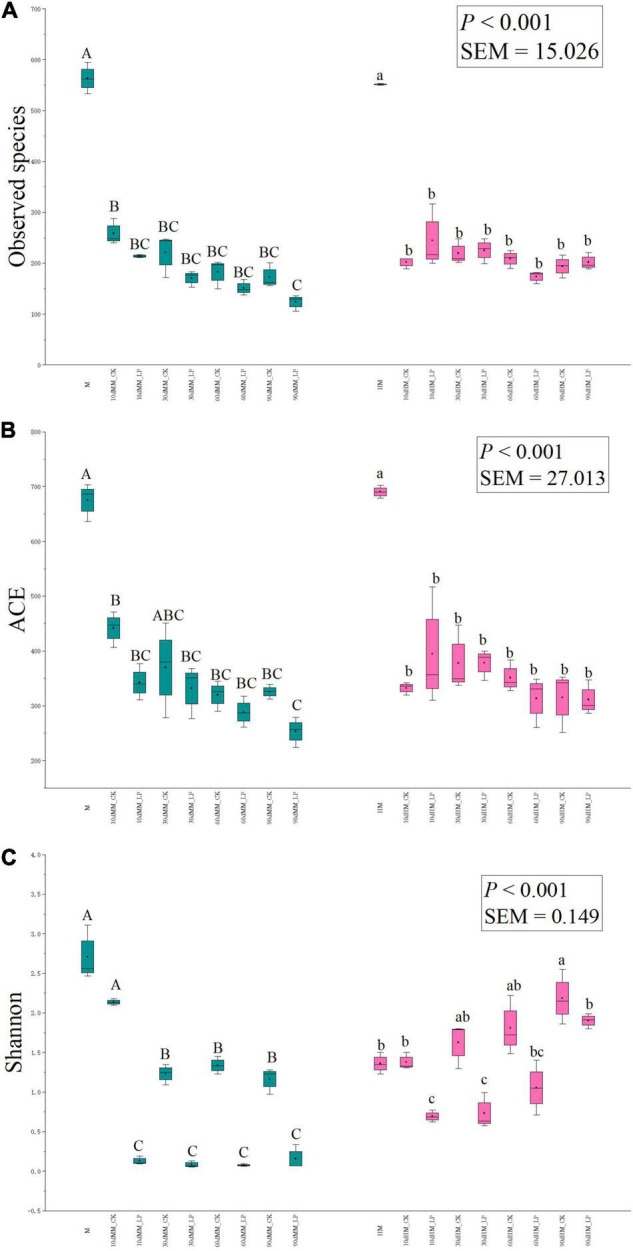
Box-plots of observed species **(A)**, ACE **(B)**, and Shannon **(C)** indices of bacterial communities in alfalfa silage at 10, 30, 60, and 90 days. HM, not wilted before ensiling; MM, wilted before ensiling; CK, silage without inoculation; LP, silage inoculated with *L. plantarum* A345. Values with different capital letters indicate significant difference among samples in the wilted group (*P* < 0.05); Values with different small letters indicate significant difference among samples in the non-wilted group (*P* < 0.05). SEM, standard error of means.

The richness of bacterial communities in silage, as indicated by observed species and Abundance-based Coverage Estimator (ACE) indices, and the diversity, as indicated by Shannon indices, are shown in Figure 4. As compared with the direct-cut alfalfa, the Shannon index increased after wilting (*P* < 0.05). The observed species and ACE indices were reduced in the ensiled as compared to the non-ensiled alfalfa in both the wilted and non-wilted groups. Inoculation of *L. plantarum* decreased the Shannon index of the wilted silage as compared with the CK group throughout the entire ensiling process. As for the non-wilted silage, although the Shannon index of the inoculated group was decreased as compared to the non-inoculated silage at 10 (*P* < 0.001) and 30 days (*P* = 0.01), differences between the two treatments were not significant at 60 (*P* = 0.07) and 90 days (*P* = 0.28).

**FIGURE 5:**
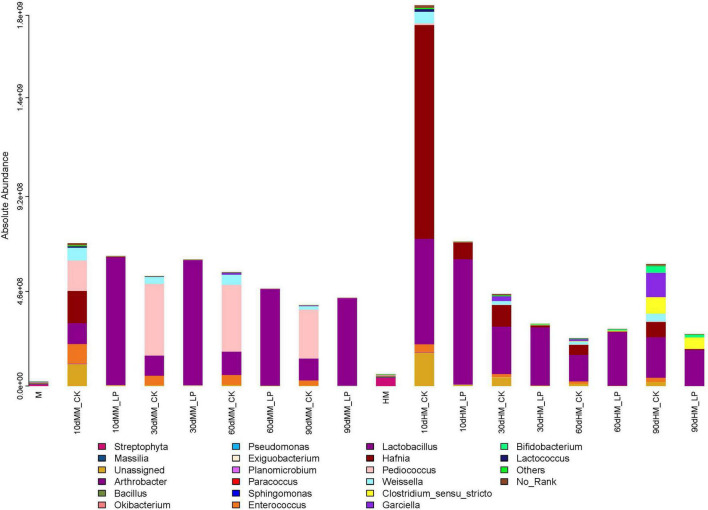
Bar plots of bacterial community structures in alfalfa silage at 10, 30, 60, and 90 days. Genus with proportions in bacterial community below 1% were combined as “Others.” HM, not wilted before ensiling; MM, wilted before ensiling; CK, silage without inoculation; LP, silage inoculated with *L. plantarum* A345.

**FIGURE 6:**
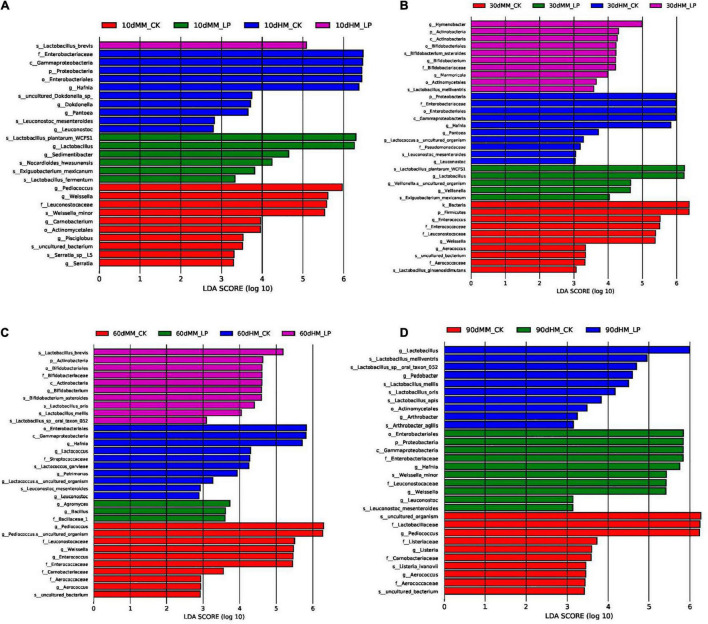
Comparison of microbial variations at 10 **(A)**, 30 **(B)**, 60 **(C)**, and 90 **(D)** days using LEfSe analysis. HM, not wilted before ensiling; MM, wilted before ensiling; CK, silage without inoculation; LP, silage inoculated with *L. plantarum* A345.

As shown in Figure 5, the total abundance of the bacterial community in fresh alfalfa decreased after wilting (7.83 × 10^7^ vs. 2.21 × 10^7^ copies/ng DNA). Inoculation of *L. plantarum* changed the dominant genus of the bacterial community in the wilted silage. *Lactobacillus* rapidly dominated the bacterial community at 10 days and remained dominant afterward in the MM_LP silage with a relative abundance greater than 98%. Rapid growth of *Lactobacillus* in the MM_LP silage at 10 days was also confirmed by LEfSe analyses (Figure 6A). At 10 days, the MM_CK silage had a high bacterial diversity, although *Pediococcus* gradually dominated with a relative abundance greater than 60%. Significance in abundance of *Pediococcus* at 10, 60, and 90 days in the MM_CK silage was also illustrated by LEfSe analyses (Figures 6A,C,D). LEfSe analyses also demonstrated that the abundance of *Weissella* was significantly greater at 10, 30, and 60 days in the MM_CK silage, as compared with the other treatment groups (Figures 6A–C).

The growth of *Hafnia* was relatively rapid in the HM_CK silage, reaching a relative abundance of 55.31% at 10 days, as also confirmed by LEfSe analyses. *Lactobacillus* dominated the bacterial community after inoculation and effectively inhibited the growth of *Hafnia* at 10 days (1.57 × 10^8^ vs. 2.38 × 10^6^ copies/ng DNA). The abundances of *Garciella*, *Clostridium sensu stricto*, and *Bifidobacterium* were detected in the HM_CK silage at 90 days. Inoculation of *L. plantarum* decreased the abundances of *Garciella* and *Bifidobacterium* at 90 days in the non-wilted silage as compared with the CK group (*P* < 0.05), but had little effect on growth of *Clostridium sensu stricto* (*P* = 0.71).

### Spearman’s Correlation Analyses of Fermentation Properties and Bacterial Communities

Correlations of fermentation properties with abundance of the top 10 most abundant genera in bacterial communities in the non-inoculated and inoculated silage are shown in Table 2. In the non-inoculated silage, *Pediococcus* and *Enterococcus* were negatively correlated with AA and NH_3_-N (*P* < 0.05). Meanwhile, NH_3_-N was positively correlated with *Lactobacillus*, *Garciella*, *Clostridium sensu stricto*, and *Bifidobacterium* (*P* < 0.05) in the non-inoculated silage. AA was positively correlated with *Lactobacillus*, *Clostridium sensu stricto*, and *Bifidobacterium* (*P* < 0.05), and negatively correlated with *Weissella* (*P* < 0.05) in the non-inoculated silage. The pH had positive correlations with *Hafnia*, and LA-producing genera *Weissella* and *Lactococcus* (*P* < 0.05) in the non-inoculated silage. Besides, positive correlations of PA with *Garciella*, *Clostridium sensu stricto*, and *Bifidobacterium*, and BA with *Clostridium sensu stricto* and *Bifidobacterium* were performed in the non-inoculated group (*P* < 0.05). Poor correlations of LA with these genera were performed in the non-inoculated silage (*P* > 0.05).

**TABLE 2:** Spearman’s correlation analyses of fermentation properties with abundance of the top 10 most abundant genera in the non-inoculated and inoculated silage^1^.

	pH		LA		AA		PA		BA		NH_3_-N	
	*r*	*P*	*r*	*P*	*r*	*P*	*r*	*P*	*r*	*P*	*r*	*P*
***Correlations of fermentation properties with abundance of the top 10 abundant genus in the non-inoculated silage***												
*Lactobacillus*	0.310	0.140	0.147	0.493	0.574	0.003	0.286	0.175	0.274	0.195	0.463	0.023
*Hafnia*	0.699	<0.001	–0.022	0.920	0.294	0.163	0.046	0.830	0.048	0.822	0.214	0.316
*Pediococcus*	–0.156	0.467	–0.197	0.355	–0.796	<0.001	–0.215	0.313	–0.312	0.138	–0.737	<0.001
*Unassigned*	0.744	<0.001	–0.089	0.680	0.243	0.253	0.130	0.546	0.104	0.627	0.216	0.312
*Enterococcus*	0.316	0.133	–0.316	0.133	–0.716	<0.001	–0.114	0.595	–0.241	0.258	–0.584	0.003
*Weissella*	0.545	0.006	–0.267	0.207	–0.417	0.042	0.258	0.223	0.150	0.485	–0.216	0.312
*Garciella*	–0.110	0.609	0.050	0.818	0.243	0.252	0.480	0.018	0.242	0.255	0.453	0.026
*Clostridium* ^2^	0.336	0.108	0.108	0.616	0.697	<0.001	0.480	0.018	0.575	0.003	0.790	<0.001
*Bifidobacterium*	0.021	0.923	0.150	0.485	0.759	<0.001	0.478	0.018	0.557	0.005	0.842	<0.001
*Lactococcus*	0.510	0.011	0.120	0.576	–0.148	0.491	–0.034	0.876	–0.088	0.684	0.049	0.821
***Correlations of fermentation properties with abundance of the top 10 abundant genus in the inoculated silage***												
*Lactobacillus*	–0.467	0.021	0.510	0.011	–0.600	0.002	–0.346	0.097	–0.574	0.003	–0.675	<0.001
*Hafnia*	0.732	<0.001	0.426	0.038	0.664	<0.001	0.015	0.944	0.149	0.487	0.599	0.002
*Pediococcus*	0.405	0.050	0.541	0.006	0.313	0.137	0.075	0.727	0.032	0.883	0.344	0.100
*Unassigned*	0.531	0.008	0.648	0.001	0.375	0.071	0.015	0.944	–0.060	0.781	0.222	0.298
*Enterococcus*	0.094	0.664	0.674	<0.001	–0.061	0.776	–0.286	0.175	–0.431	0.036	–0.134	0.534
*Weissella*	0.050	0.815	0.634	0.001	–0.170	0.428	–0.286	0.175	–0.570	0.004	–0.354	0.089
*Garciella*	–0.239	0.262	0.610	0.002	–0.467	0.021	–0.136	0.528	–0.454	0.026	–0.538	0.007
*Clostridium* ^2^	0.462	0.023	0.044	0.837	0.470	0.021	0.346	0.097	0.578	0.003	0.391	0.059
*Bifidobacterium*	0.644	0.001	–0.236	0.268	0.756	<0.001	0.346	0.097	0.470	0.020	0.812	<0.001
*Lactococcus*	0.745	<0.001	0.298	0.157	0.734	<0.001	0.196	0.359	0.180	0.400	0.634	0.001

*^1^LA, lactic acid; AA, acetic acid; PA, propionic acid; BA, butyric acid; NH_3_-N, ammoniacal nitrogen.*

*^2^Clostridium, Clostridium sensu stricto.*

Spearman’s correlation analysis of the relationships among the fermentation properties and bacterial genera in the inoculated silage showed that *Lactobacillus* was positively correlated with LA and negatively correlated with pH, AA, BA, and NH_3_-N (*P* < 0.05). Meanwhile, pH were positively correlated with *Hafnia*, *Clostridium sensu stricto*, *Bifidobacterium*, and the LA-producing genera *Pediococcus* and *Lactococcus* (*P* < 0.05) in the inoculated silage. LA had positive correlations with *Hafnia*, *Pediococcus*, *Enterococcus*, *Weissella*, and *Garciella* (*P* < 0.05) in the inoculated silage. AA and NH_3_-N were positively correlated with *Hafnia*, *Clostridium sensu stricto*, *Bifidobacterium*, and *Lactococcus*, and negatively correlated with *Garciella* (*P* < 0.05) in the inoculated silage. NH_3_-N was also negatively correlated with *Weissella* in the inoculated group (*P* < 0.05). BA was positively correlated with *Clostridium sensu stricto* and *Bifidobacterium* (*P* < 0.05) in the inoculated silage.

## Discussion

### Effects of Wilting Before Ensiling on the Fermentation Dynamics of Alfalfa Szertilage

The moisture content of alfalfa plays an important role in the ensiling process. The optimal DM content of wilted alfalfa to prevent effluent production is reportedly 300–400 g/kg FW (Duniere et al., 2013). The reduction in WSC concentration after wilting was consistent with previous reports by Tao et al. (2017) and Agarussi et al. (2019). The WSC concentrations of both the wilted and non-wilted alfalfa were greater than 50 g/kg DM, which is considered sufficient for adequate fermentation during ensiling (Ni et al., 2018). Similar observed species and ACE indices of the bacterial communities of wilted and non-wilted alfalfa indicated that wilting had only slight effects on richness of bacterial community. A reduction in total bacterial abundance and increase in the Shannon index of the wilted alfalfa indicated that wilting mainly altered the abundances of bacterial species in alfalfa. The richness of bacterial communities was reduced by ensiling of alfalfa regardless of wilting, which is consistent with a previous study by Keshri et al. (2018) of corn silage, indicating that ensiling inhibited the growth of some epiphytic microorganisms.

Ammoniacal nitrogen is recognized as a marker of proteolytic activity (Scherer et al., 2015). The growth of *Hafnia* was inhibited in the wilted silage at 10 days as compared with the non-wilted silage both in the CK and inoculated groups. The genus *Hafnia*, a member of the family *Enterobacteriaceae*, consists of species that transform nitrogen in silage into alkaline products, such as biogenic amines and other NH_4_^+^-containing compounds, which can explain the reduction in NH_3_-N accumulation in the wilted silage as compared with the non-wilted silage. Plant proteolytic enzymes also promote the accumulation of NH_3_-N (Yang et al., 2020). As reported by Tao et al. (2012), the activities of most plant proteolytic enzymes are increased at pH 5.0–6.0 in alfalfa silage, which can explain the higher concentration of NH_3_-N in HM_CK silage at 10 days as compared with other treatments. Comparisons of the two non-inoculated silages at 10 days indicated that wilting delayed pH decline and LA accumulation in silage. The wilted silage also contained higher concentrations of WSC, glucose, and fructose at 10 days as compared with the non-wilted silage, possibly because of reduced activities of some microorganisms, including LA-producing bacteria, due to the change of osmotic stress caused by wilting in the wilted silage (Wang et al., 2019). Change of the osmotic stress in alfalfa might affect activities of microorganisms.

The abundances of epiphytic *Lactobacillus* and *Pediococcus* decreased after wilting (2.82 × 10^5^ vs. 1.22 × 10^5^ copies/ng DNA and 4.00 × 10^4^ vs. 1.75 × 10^4^ copies/ng DNA, respectively), which presents another possible cause of the delay in LA fermentation. Although the pH of the wilted silage was higher than that of non-wilted silage at 10 days, prolonged ensiling further decreased the pH to a similar level as the non-wilted silage at 30 days. At 90 days, the pH of the HM_CK silage was increased and significantly greater than that of the MM_CK silage (*P* < 0.05). Similar dynamics of pH in wilted and non-wilted alfalfa silage were also reported by Agarussi et al. (2019). The major bacteria involved in LA fermentation of alfalfa silage belong to the genera *Lactobacillus*, *Pediococcus*, *Weissella*, and *Leuconostoc* (Pang et al., 2011; Ni et al., 2018). Wilting caused a change in the major genus involved in LA fermentation in the non-inoculated silage during ensiling from *Lactobacillus* to *Pediococcus*. *Pediococcus* species grow rapidly during ensiling when the pH is between 5 and 6.5 (McDonald et al., 1991; Kung et al., 2003). The change in pH allowed the predominance of *Pediococcus* in the MM_CK silage. Meanwhile, *Lactobacillus* became the major genus involved in LA fermentation in HM_CK silage, indicating that *Lactobacillus* might be more competitive than *Pediococcus* under high moisture conditions as compared with the wilted group.

Clostridia fermentation occurred in the non-wilted silage at 90 days, as indicated by the increased pH and growth of genera belonging to the class Clostridia, such as *Clostridium sensu stricto* (Pahlow et al., 2003). This also led to LA consumption and accumulation of weaker volatile fatty acids like PA and BA. A sharp reduction in the galactose concentration was observed in the non-wilted silage at 90 days as compared with 60 days (*P* < 0.05) along with Clostridia fermentation, suggesting a potential correlation of galactose consumption with the growth of clostridia species. Hence, further research is warranted to explore the role of galactose in Clostridia fermentation in silage. Fermentation quality remained stable from 30 to 90 days in the wilted silages, which might have been due to the lower moisture content of the wilted silage that limited the activities of microorganisms, resulting in greater stability of the bacterial community.

### Effects of *Lactobacillus plantarum* on Fermentation Dynamics of Alfalfa Silage

*Lactobacillus plantarum* is reported to improve the fermentation quality of alfalfa silage (Ogunade et al., 2018; Yang et al., 2019; Zhao et al., 2020). In this study, *L. plantarum* inoculation improved the fermentation qualities of both the wilted and non-wilted alfalfa silages, as indicated by the greater decreases in pH and NH_3_-N accumulation.

*Lactobacillus plantarum* inoculation of the non-wilted silage reduced the diversity of the bacterial community at 10 and 30 days and inhibited the growth of *Hafnia* as compared with the HM_CK silage. This finding was consistent with the reduction in NH_3_-N accumulation in the inoculated silage as compared with the CK group. Lindgren et al. (1990) reported that *L. plantarum* metabolizes LA to AA under sugar-deficient conditions, which can explain the reduction in LA and increase in AA accumulation at 60 days compared with 30 days in the HM_LP silage. *L. plantarum* inoculation led to an increase in the AA content because of prolonged ensiling, similar to the report by Parvin and Nishino (2009) of guinea grass silage. *L. plantarum* inoculation prevented the apparent increase in pH of the non-wilted silage at 90 days as compared with the CK group (*P* < 0.05) and also inhibited BA formation and growth of *Garciella* and *Bifidobacterium* species at 90 days as compared with the HM_CK silage (*P* < 0.05). These results indicate that inoculation of *L. plantarum* weakened Clostridia fermentation by inhibiting the growth of *Garciella*.

In the wilted silage, *L. plantarum* inoculation accelerated LA fermentation as indicated by the rapid decrease in pH, LA accumulation, and dominance of *Lactobacillus* at 10 days (*P* < 0.05). An apparent abundance of *Arthrobacter* was observed in the MM_CK silage at 10 days. *Arthrobacter* is commonly found on the aerial surfaces of plants with highly proteolytic activities (Gobbetti and Rizzello, 2014). Inhibition of *Arthrobacter* in the inoculated silage at 10 days indicated that activities of plant proteolytic enzymes might be reduced by *L. plantarum* inoculation, in agreement with the reduced concentration of NH_3_-N in the inoculated silage as compared with the MM_CK group at 10 days (*P* < 0.05). Inoculation also reduced the diversity of the bacterial community and altered the dominant genera in wilted alfalfa silage as compared with the CK group. These results indicate that *L. plantarum* inoculation promoted adaption to the ensiling conditions and, thus, is considered more competitive than epiphytic microorganisms.

### Correlations of Fermentation Properties With Bacterial Communities in Alfalfa Silage

Correlations between the bacterial genera and fermentation properties in the non-inoculated and inoculated silage were demonstrated. In the non-inoculated group, *Pediococcus* was the most abundant genus involving in LA fermentation in the wilted silage. Apparent abundance of *Enterococcus* was also observed in the non-inoculated silage during ensiling, and higher abundance of *Enterococcus* was observed in the wilted silage compared with the non-wilted group at 10, 30, and 60 days. These results were in accordance with the negative correlations with NH_3_-N of the two genera. *Lactobacillus* was the predominant genus involving in LA fermentation in the non-wilted CK silage, while it failed to stabilize the bacterial community and achieve adequate LA fermentation. The negative correlation of *Lactobacillus* with fermentation quality might be resulted from the poorer fermentation quality of the non-wilted silage compared with the wilted silage in the non-inoculated group. *Weissella* is a heterofermentative genus, and is considered sensitive to lower pH in silage (Yang et al., 2019). This might explain its positive correlation with pH in the non-inoculated silage, as it generally performed higher abundance in the silage at early stage of ensiling. The negative correlation of *Weissella* with AA might be resulted from activities of other AA producers in the non-inoculated silage. Apparent abundance of *Lactococcus* was observed in the non-inoculated silage at 10 days both in the wilted and non-wilted group, and was outcompeted by other microorganisms with prolonged ensiling process. This was in accordance with its positive correlation with pH in silage.

In accordance with previous studies by Ogunade et al. (2018) and Yang et al. (2019), correlation analyses indicated positive effects of *Lactobacillus* on fermentation properties in alfalfa silage, including decreased pH, LA accumulation, and inhibition of BA and NH_3_-N production. *Lactobacillus* is considered a main producer of LA in silage and plays an important role in decreasing pH and inhibiting growth of proteolytic microorganisms during ensiling (Cai et al., 1998). However, *L. plantarum* inoculation had little effect on the growth of *Clostridium sensu stricto* in the non-wilted silage. Thus, *Clostridium sensu stricto* might be a major BA-producer in the HM_LP silage at 90 days. Other LA-producing genera, including *Pediococcus*, *Enterococcus*, *Weissella*, and *Lactococcus*, performed low proportions in bacterial community in the inoculated silage (<0.2%), and abundance of these genera decreased with prolonged ensiling process. Positive correlation of LA-producing cocci with pH in silage is consistent with that of previous studies by Ogunade et al. (2018), Yang et al. (2019), and Dong et al. (2020). Abundant LA accumulation was performed at 10 days both in the wilted and non-wilted silage in the inoculated group. This might explain the positive correlations of *Pediococcus*, *Enterococcus*, and *Weissella* with LA in the inoculated silage. Although *Leuconostoc* is also considered as a major genus involved in LA fermentation during ensiling, it performed low abundance in the silages in this study with the proportions in bacterial communities below 1%.

The abundance of *Hafnia* was higher in the non-wilted silage at 10 days compared with the wilted group along with higher NH_3_-N accumulation. Correlation analyses also illustrated its positive correlation with pH both in the non-inoculated and inoculated silage, and positive correlation with NH_3_-N concentration in the inoculated group. *Garciella* and *Clostridium sensu stricto* were negatively correlated with fermentation quality in the non-inoculated silage, which was predictable, because both genera are considered to participate in Clostridia fermentation. However, there was a slight correlation between *Garciella* and BA (*P* > 0.05) in the non-inoculated group, as an abundance of *Garciella* was also observed in MM_CK silage at 60 days (8.65 × 10^6^ copies/ng DNA) and HM_CK silage at 30 days (2.27 × 10^7^ copies/ng DNA), while no accumulation of BA was detected in these silages, which may have resulted from the activities of microbes that utilize BA in silage (Yang et al., 2020). The gene expression patterns of species in a bacterial community are strictly regulated by both time and initial structures, and the major species have a greater impact on the gene expression profiles of minor species than vice versa (Gao et al., 2021). The duration of ensiling and abundances of other species in the bacterial community might affect the gene expression profiles of *Garciella* in silage. *Garciella* had positive correlation with LA, and negative correlations with AA, BA, and NH_3_-N in the inoculated silage. Proportion of *Garciella* was below 1% in the inoculated silage. The positive correlation of *Garciella* with fermentation quality in the inoculated silage might be resulted from the relatively higher abundance of *Garciella* in the wilted silage compared with the non-wilted group. The abundance of *Bifidobacterium* increased along with growth of *Clostridium sensu stricto* in the non-wilted silage at 90 days as compared with 60 days. An abundance of *Bifidobacterium* was also reported in direct-cut alfalfa silage after Clostridia fermentation (Zheng et al., 2017), although the role of this genus in alfalfa ensiling remains unclear. This anaerobic genus might be able to adapt well to the conditions created by Clostridia fermentation in silage.

Wilting of fresh alfalfa to an appropriate moisture content before ensiling could largely improve the fermentation quality of alfalfa silage (Tao et al., 2017; Agarussi et al., 2019). However, climatic conditions, such as high precipitation, can delay wilting of ensiling material to an optimum moisture content (Yang et al., 2020). In this study, the high moisture content of the silage led to faster consumption of WSCs and growth of some undesirable microorganisms, such as *Hafnia*, at 10 days, which further led to insufficient LA fermentation in the silage and BA was observed at 90 days. Although *L. plantarum* inoculation improved fermentation quality in the non-wilted silage, there was little effect against the growth of *Clostridium sensu stricto* in this study. These *Clostridium sensu stricto* strains need to be isolated and inoculation of LAB with high antibacterial activities against these strains should be further analyzed. Besides, the addition of acids along with *L. plantarum* could also aid in the inhibition of activities of plant proteolytic enzymes and growth of undesirable microorganisms in the early stage of alfalfa ensiling.

## Conclusion

Fermentation properties and microbiome results indicated that the high moisture content of the non-inoculated silage led to faster consumption of WSCs and rapid growth of some undesirable microorganisms in the early stage of ensiling, which promoted Clostridial fermentation. Wilting and *L. plantarum* inoculation both improved fermentation quality and inhibited the growth of spoilage microorganisms in alfalfa silage to an extent, while inoculation of *L. plantarum* alone failed to stabilize the bacterial community and achieve optimum fermentation quality of non-wilted alfalfa silage.

## Data Availability Statement

The datasets presented in this study can be found in online repositories. The names of the repository/repositories and accession number(s) can be found below: https://www.ncbi.nlm.nih.gov/, PRJNA773516.

## Author Contributions

FY: conceptualization, formal analysis, writing–original draft, and visualization. YW: writing–review and editing, supervision, project administration, and funding acquisition. SZ: methodology, formal analysis, validation, and investigation. CF: funding acquisition and resources. XF: resources and methodology. All authors contributed to the article and approved the submitted version.

## Conflict of Interest

The authors declare that the research was conducted in the absence of any commercial or financial relationships that could be construed as a potential conflict of interest.

## Publisher’s Note

All claims expressed in this article are solely those of the authors and do not necessarily represent those of their affiliated organizations, or those of the publisher, the editors and the reviewers. Any product that may be evaluated in this article, or claim that may be made by its manufacturer, is not guaranteed or endorsed by the publisher.

## Footnotes

1
https://github.com/dstreett/FLASH2

2
https://drive5.com/uparse/

3
https://mothur.org/wiki/mothur_v.1.9.0/

4
https://www.python.org/

5
http://www.r-project.org
